# Facilitators and barriers to professional nurses implementing integrated services in urban primary health care clinics in Kavango East region, Namibia

**DOI:** 10.4102/phcfm.v14i1.3604

**Published:** 2022-12-19

**Authors:** Daniel O. Ashipala, Joseph Himarwa

**Affiliations:** 1 Department of General Nursing Sciences, Faculty of Health Sciences and Veterinary Medicine, University of Namibia, Rundu, Namibia; 2 Department of General Nursing Sciences, Faculty of Health Sciences and Veterinary Medicine, University of Namibia, Windhoek, Namibia

**Keywords:** facilitators, barriers, implementation, person-centred model, integrated services, urban primary health care, professional nurses

## Abstract

**Background:**

The World Health Organization (WHO) has advocated for the implementation of people-centred and integrated health services. Although there is growing evidence of integration’s benefits for sexual and reproductive health, human immunodeficiency virus (HIV) and rights, health services face tremendous resource constraints when it comes to integrating these services.

**Aim:**

The aim of study was to explore and describe the facilitators and barriers to professional nurses implementing the person-centred model of integrated services in urban primary health care clinics in the Kavango East region, Namibia.

**Setting:**

We interviewed professional nurses from urban primary health care clinics in low-resourced settings in Rundu health district, Kavango East region, Namibia.

**Methods:**

This study employed a qualitative approach utilising an explorative, descriptive and contextual strategy. Semistructured interviews were used to collect the data. Fifteen participants were selected using a purposive sampling technique. The interviews were audio recorded, transcribed verbatim and coded, before the data were analysed using thematic analysis.

**Results:**

The data analysis led to the emergence of the following four themes: understanding integrated services; facilitators for implementing integrated services; barriers to the implementation of integrated services; and improvement measures for implementing integrated services.

**Conclusion:**

Findings showed that the implementation of integrated services faces many barriers, which are related to lack of human resources skills, a lack of essential supplies and space constraints. These findings will hopefully create an awareness and understanding of the facilitators and barriers that professional nurses face in the implementation of integrated services for urban primary health care in the Namibian urban context.

**Contribution:**

The study’s findings can be used to develop strategies and ongoing interventions that focus on addressing the barriers professional nurses face in the implementation of integrated services in both urban and rural primary health care settings.

## Introduction

The World Health Organization’s (WHO) global strategy on people-centred, integrated health services is a call for a fundamental paradigm shift in the way that health services are funded, managed and delivered.^1^ Integration is a complex process that can be conceptualised in two ways,^2^ that is, horizontal and vertical. Horizontal integration involves linking similar levels of care, while upright integration links different levels of care.^3^ The idea of integrated health services is not new; patient-centred care (PCC) was introduced in the 1970s^4^ and was the basis for the focus on primary health care (PHC) in the 1980s. However, in the 1980s, a selective PHC was encouraged with a focus on specific high impact interventions.^5^ In the 1990s, because of the human immunodeficiency virus (HIV) emergency and earmarked donor funding, a ‘vertical’ approach to provide health services was implemented.^5^ This is the most logical way to organise health services and the only way that does not compromise universal access to a broad range of services.^6^

Health services face resource constraints in integrated services, particularly human resource shortages.^6^ A better understanding of the barriers and facilitators to PCC could improve its delivery.^7^ Previous models of PCC and studies identifying the barriers and facilitators to implementation focused on expert opinions.^8^ WHO defines health service integration as a way to ensure that people receive a continuum of care according to their needs throughout their lifetimes. The Ministry of Health and Social Services in Namibia has implemented a model of care on ‘integrated people-centred health services’ as a window of opportunity to integrate health services.^1^

Namibia has a generalised, mature HIV epidemic with an HIV prevalence of 12.6%^8^ and high antiretroviral (ARV) coverage (77.4%).^8^ From a sexual reproductive health perspective, there is high coverage of antenatal care (97.0%), skilled deliveries (88.0%) and modern contraceptive use (50.0%).^9^ However, the country continues to experience unacceptably high levels of maternal mortality (200/100 000), teenage pregnancies (18.6%) and unmet family planning (FP) needs (15.0%).^8^ Sexual reproductive health, HIV and other related services, such as the integrated management of neonatal and childhood illnesses, the integrated management of pregnancy and childbirth and the integrated management of adolescent and adult illness in PHC facilities in Namibia, have limited integration in health service delivery.^6^ Analysis from the ‘pre’ baseline assessment^9^ showed that health services in the Rundu health district facilities are provided according to a nonintegrated approach and operate as two different clinics (PHC and ARV), with very limited interaction and coordination between them. Not all PHC services are provided on a daily basis. This poses a challenge for accessibility, because integrated services in Namibia are provided in a resource-constrained setting.^6^ This is not surprising, as such programmes have been argued to potentially improve patient care and more efficiently allocate scarce resources.^10^

The barriers and facilitators to professional nurses implementing integrated services have been explored in developed countries; however, no such studies in Namibia have been documented. It is for this reason that the researchers of this study seek to explore the facilitators and barriers to professional nurses implementing integrated services at urban PHC facilities in Namibia. The findings from this study will hopefully create awareness and understanding of this topic in the Namibian urban context and will recommend the measures needed to improve the implementation of integrated services in Namibian PHC clinics.

## Research aim

The aim of this study was to explore and describe the facilitators and barriers to professional nurses implementing integrated services in urban PHC clinics in Kavango East region, Namibia.

### Framework of the study

**FIGURE 1:**
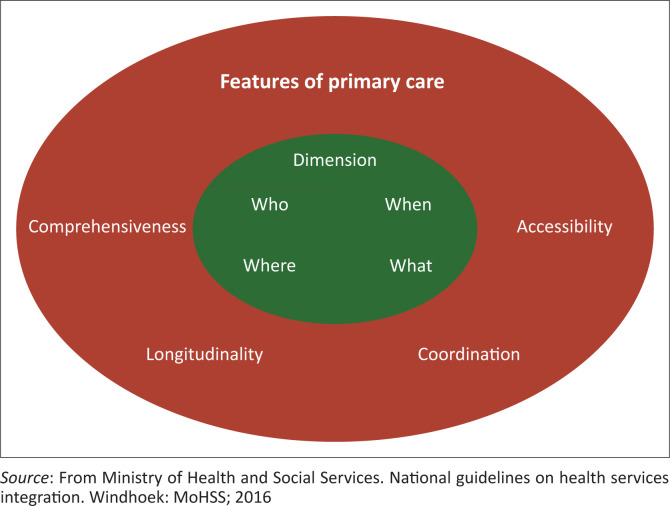
Integration model of integrated services for primary health care facilities in Namibia.^6^

The current study was based on the Namibia integration model (Figure 1), also known as the main four features of primary care. In this model, a nurse will always sit in the consultation room and provide care to the same patient over time. The theory incorporates four features of primary care: accessibility, comprehensiveness, continuity or longitudinality and coordination. The first feature is accessibility, which is the first contact, access and use by addressing geographical, administrative, cultural and time barriers. The second feature, comprehensiveness, ensures that the nurse has all their needs catered for in the consulting room (register, equipment, medicines, etc.) in order to provide all the services that the patient needs within the same room. The third feature, continuity or longitudinality, sees the same nurse following the same patient over time and assuming responsibility for that patient. The last feature in this model is coordination among health care teams across different levels of the health care system. This model assumes a one-stop health care approach, with flexible facilitated referrals within and outside a facility.^6^ In this study, the Namibian integrated model theory was important to support the results, discussions, conclusions and recommendations of the study.

## Research methods and design

A qualitative, explorative, descriptive and contextual research design was utilised^11^ in order to allow the researcher to gain more information on the phenomena under study.^12^ According to Grove et al.,^13^ the goal of explorative research is to understand the underpinnings of specific phenomena and explain specific and systematic relationships among them, so that they are described in rich detail. According to Maree,^14^ qualitative research design is naturalistic, focusing on natural settings where interactions occur. In addition, it is used to explore how people make sense of their surroundings, experiences and understandings of phenomena.^15^ The chosen approach and design enabled the researchers to explore and describe the experiences of professional nurses with regard to the facilitators and barriers to implementing integrated services in the urban PHC facilities of the Kavango East region, Namibia.

### Context

The current study was conducted at five urban PHC facilities within Rundu health district in the north-east of Namibia. The integrated health services at these clinics focus mainly on sexual and reproductive health, HIV rights and related health services such as the integrated management of neonatal and childhood illnesses, wound dressing, antenatal and postnatal care, history taking, antiretroviral therapy, point of service assessment, HIV counselling and testing, emergencies, immunisations, FP and the integrated management of adolescent and adult illnesses in PHC facilities.^6^ The total number of professional nurses at these clinics ranges between 5 and 10. Patients seen per day are estimated at between 300 and 400; that is, each nurse sees approximately 58 – 60 patients a day. Common ailments seen in these clinics involve acute care, while the Mister Sister mobile clinics provide a range of services in line with the PHC model, including routine immunisations, diagnosis and treatment of routine communicable diseases, management of minor trauma, testing and follow-up treatment of chronic diseases, voluntary counselling and testing for HIV, antenatal care and health education.^16^

### Recruitment, sample and sampling technique

The study population comprised 15 professional nurses who were working at the five selected integrated PHC clinics in Rundu district. This study utilised a purposive nonprobability sampling technique, with the researchers selecting study participants who would provide rich data on the phenomenon of interest.^17^ This was achieved by purposively selecting only those professionals who met the inclusion criteria for the study. In order to be selected for this study, nurses needed: (1) to be a professional nurse, registered with the Namibia Nursing Council (NNC) and (2) to have provided integrated health care services in urban clinics in the Rundu health district for at least one year. Data were collected until saturation was achieved with the 15th participant.

### Data collection

Data in this study were collected in August and September 2021. Participants were approached by the researcher, who explained the aim of the study, after which the participants who agreed to participate were asked to sign a consent form.^18^ Prior to data collection, a pilot test was conducted on two professional nurses with the aim of refining the interview guide. The pilot interview lasted for 30 – 40 min, and no changes were made to the guide. Findings of the pilot test were included in this study. The date, time and place of each interview were confirmed with the participants. Data were collected using semistructured interviews in accordance with an interview guide that was developed based on the research questions and the study objectives, as well as the literature review. The researcher made use of field notes to record observed nonverbal cues and body language. The interviews were conducted by the researcher in person at a location most convenient to the participant and were on average 30 – 35 min long. The researchers transcribed the interviews verbatim before integrating their field notes. Data saturation was reached with the 15th participant, after which no new information emerged. The researcher utilised a list of guiding questions to prompt any response or further description of answers where required:

- What are the facilitators to professional nurses implementing integrated services in urban PHC clinics in Kavango East region, Namibia?
- What are the barriers to professional nurses implementing integrated services in urban PHC clinics in Kavango East region, Namibia?
- What recommendation can be made to support the implementation of integrated services in urban PHC clinics in Kavango East region, Namibia?

### Data analysis

The researchers transcribed the interviews verbatim before integrating their field notes. Data from the interviews were then analysed using Braun’s six-step method of qualitative data analysis; that is: (1) organise and prepare the data; (2) develop a sense of all the data; (3) code the data following Tesch’s nine steps; (4) identify and describe themes; (5) represent the findings; and (6) interpret the data.^19^ The data were subsequently analysed by the researchers and an independent coder experienced in qualitative research to give meaning.^20^ They then held a consensus discussion and agreed on the main themes and subthemes that emerged. The researchers are academic members of a Namibian university; currently they are no longer involved in service provision. They are experienced in qualitative research and are consultants who supervise undergraduate and postgraduate degrees. A field journal was used to reflect researcher behaviours and experience.

### Measures to ensure trustworthiness

Trustworthiness or rigour refers to the degree of confidence in the data, interpretation and methods used to ensure the quality of a study.^14^ To ensure rigour, the study adhered to the criteria as outlined by Lincoln and Guba,^21^ that is, credibility, dependability, confirmability and transferability.

Credibility refers to the truthfulness of the data.^14^ In this study, credibility was ensured through data analysis that truly reflected the data collected and prolonged engagement (the researcher stayed in the field for one month until data saturation was reached to gain an in-depth understanding of the phenomenon). Triangulation was ensured by the researcher taking field notes in order to observe verbal and nonverbal communication. In order to achieve dependability, data were confirmed with an independent coder, who was experienced in qualitative research and held a doctorate, in a consensus meeting. In addition, the promoter of the study double-checked the transcripts to ensure consistency regarding the research questions, objectives and the entire research process.

Confirmability is the potential for data congruence in terms of accuracy, relevance or meaning. In this study, confirmability was ensured by obtaining informed consent from the participants after they were afforded the time to review the information letter. Their questions were also answered truthfully, and their freedom of expression was encouraged. Additionally, verbatim transcription of the interviews and rereading of these and the field notes enabled the researcher to get a better understanding of what the participants said about integrated services. According to Pescheny et al.,^22^ transferability essentially refers to the generalisation of data. Therefore, in this study, transferability was ensured through data saturation, purposive sampling, a thick description of the study design and methods and making the findings available with supporting quotes from the participants.

### Ethical considerations

The research was conducted after approval was granted by the University of Namibia’s School of Nursing Research and Ethics Committee of the Faculty of Health Sciences (ref. no. SoNREC 9/2021), the School of Nursing Ethic and Review Committee (ref. no. SoNERC 08/2021) and the Ministry of Health and Social Services (ref. no. JKH2021). Participation in this study was voluntary, and participants were informed that they were free to withdraw from the study at a given time with no repercussions. The researcher ensured that no data was linked to any participant, with participants being referred to by a number instead of their real name.

## Findings and discussion

### Participants’ characteristics

All participants were under 50 years of age, with the majority being between the ages of 31 and 40. The sample consisted of seven male and eight female nurses who were recruited from different clinics within Rundu district. Table 1 gives more information regarding the demographic characteristics of the participants.

**TABLE 1:** Demographic characteristics of participants.

Characteristics	Total
**Age (years)**	
21–30	3
31–40	8
41–50	4
**Gender**	
Female	8
Male	7
**Rank**	
Registered nurse	10
Senior registered nurse	5
**Years of working experience**	
1–3	2
4–7	6
8–11	4
12–15	3

### Presentation of findings and discussion

The three themes that emerged from the data analysis were: facilitators of the implementation of integrated services; barriers to the implementation of integrated services and improvement measures for implementing integrated services in Namibia. Table 2 gives a description of the themes and sub themes in the study.

**TABLE 2:** Themes and subthemes from the data analysis.

Themes	Subthemes
1. Facilitators of the implementation of integrated services	Access to the guideline on integrated services Continuity of care
2. Barriers to the implementation of integrated services	Support and supervision Limited resources Time constraints Unhappiness among patients
3. Improvement measures for the implementation of integrated services	Training for nurses who work in primary health care Recruitment for more nurses Procurement of more clinical equipment Region and district to provide support and supervision

### Theme 1: Facilitators of the implementation of integrated services

This theme reflects the experiences of nurses regarding which facilitators enable the implementation of integrated services. The two subthemes that emerged from this theme were access to the guidelines on integrated services and continuity of care.

#### Subtheme 1: Access to the guidelines on integrated services

Participants noted that it was easy for everyone to follow the guidelines that the interventions were based on. A scenario where the structure, actions, roles and responsibilities are defined in the guidelines was found to be straightforward for the nurses.

One of the participants said:

‘I think the guidelines have opened our eyes in such a way that we realised that we have been implementing integrated services in a wrong way and wasting valuable time. The guideline is clear; it tells what to do, whether to give drugs or to refer, and challenges are minimised by the guidance of the guidelines, and it feels that this has really helped us a lot.’ (P8, 47 years, male, registered nurse)

Another participant noted:

‘Even though you have your training, knowledge and skills, but the most important is the national guidelines next to you that have abreast information which guides you and that you can refer to when you are not sure of what you should do.’ (P2, 31 years, female, registered nurse)

The provision of exemplary care, as described by the findings of the current study, is in line with a study conducted in South Africa on the barriers to integrated service delivery relating to physical and mental conditions, Kathol et al.^23^ The presence of explicit rules guiding practice (e.g. guidelines on prescribing, referrals and care checklists), consistency across facilities and organisations, and mechanisms for the exchange of information and lesson-learning, also emerged as important facilitators. Moreover, research on the health system facilitators and barriers to the integration of HIV and chronic disease services by Watt et al.^24^ reflected that, in addition to satisfaction with integrated meetings, professional nurses see guidelines as being appropriate and designed for providing guidance to health care workers on the management of integrated health services.

#### Subtheme 2: Continuity of care

The participants pointed out that quality of care for patients is easily monitored, especially among those who require follow-ups, as they are treated by the same health care worker.

One of the participants said:

‘Patients don’t have to go to another room to get any other services. We make use of the supermarket approach – now they can get everything they need at once, and this help you know all your patients.’ (P1, 50 years, male, registered nurse)

Another participant added:

‘Patients, especially postnatal mothers, feel a sense of holistic care when they bring their babies for immunisation and get rendered with services such as family planning and COVID-19 vaccination in one room.’ (P4, 34 years, male, senior registered nurse)

This finding corresponds with the study conducted by Kathol et al.^23^ in South Africa on the barriers to integrated service delivery relating to physical and mental conditions. Kathol et al. found that nurses expressed job satisfaction and feelings of fulfilment when managing integrated health services, especially when a patient expressed gratitude towards them for healing them and when they observed a patient’s condition improving from being extremely ill to being cured. Moreover, this finding is aligned with that of Dlwati et al.;^25^ that is, PCC is facilitated through the process and staff continuity.

### Theme 2: Barriers to the implementation of integrated services

This theme incorporates all the aspects that nurses find to be a hindrance to the implementation of integrated services, that is, inadequate support and supervision; limited resources; time constraints and tensions with patients.

#### Subtheme 1: Support and supervision

The participants revealed that support and supervision are required, as it can enhance the delivery of effective integrated services:

‘As you know, this model is new, and there is no one coming from the regional or district office to visit us at the clinic even once or twice to see the challenges we are facing on. Workshops and trainings are only given to in-charges and nurses at the district offices who never bring the much-needed information to us, the nurses working with the patients on a daily basis.’ (P5, 30 years, female, registered nurse)

‘When the project manager or supervisors are not aware of interventions and changes because of lack of proper supervision and support, to undertake all the coordination required for the programme may result in less effective and delayed implementation and delivery of integrated services initiatives.’ (P13, 44 years, male, registered nurse)

This finding supports that of Holloway and Galvin,^18^ who noted that a lack of supervision is an impediment to providing quality services in a community. Furthermore, research by Vennedey et al.^26^ on patients’ perspectives on facilitators and barriers to PCC also identified management and/or leadership barriers as an issue, including inadequate coordination between general health workers and health specialists, inadequate support from the district medical team, lack of knowledge about system structures and work processes, inability of the health system to respond to the clients’ broader needs and challenges managing outreach services.

#### Subtheme 2: Limited resources

The participants outlined that space constraints, financial constraints, increase in workloads and staff shortages are all barriers to the implementation of integrated services:

‘The disadvantage here is that the equipments and supplies are not available in the right quality. For instance, here, we don’t have tourniquet to draw of blood, not enough beds, measuring tape and other need materials.’ (P9, 49 years, female, registered nurse)

‘Nursing staff are having low morale because they are working so hard, doing the work of two people. Workload, we have got more than 300–400 patients a day here in our clinic. It is demotivating and demoralising the staff.’ (P15, 33 years, female, senior registered nurse)

‘The space is also one of the challenges; the facility lacks space for counselling of patients. This situation makes it difficult to assess or counsel a patient in privacy. The patient becomes shy to say their problems, as there is someone getting vaccinated behind the curtains in the same room.’ (P6, 36 years, male, registered nurse)

The findings of this study are similar to those of Kathol et al.’s^23^ study on barriers to integrated service delivery relating to physical and mental conditions; they note that management needs to provide material resources such as masks, gloves and respirators in order for nurses to assess, diagnose and treat their clients effectively. Without the necessary resources, it is difficult for PHC nurses to manage all health services.

#### Subtheme 3: Time constraints

Lack of time seems to be the barrier experienced most by nurses. The results showed that not only is there not enough time to see patients, but certain procedures require a lot of time to complete, which leads to insufficient time to manage patients’ needs and wishes.

One participant said that:

‘Time that we have to treat patients feels not to be enough, so sometimes you will just rush to finish treating the patient in order for you to see others. I feel like some patients need more time than others, but the timeframe does not allow us.’ (P6, 36 years, male, registered nurse)

Another nurse added:

‘This time of 8 to 5 is not enough to cater [*for*] patients in this model, as some of the services, it can take 45 min – even up to 1 h – to see just one patient, so a requesting for over-time or time to be extended, even 7 to 7 and so, because this time is really not enough for integrated services.’ (P8, 47 years, male, registered nurse)

This finding is similar to a study by Moore et al.,^27^ who reported that there is insufficient time to provide patient-centred service delivery. Alharbi^28^ also mentioned that sufficient time is crucial when implementing something new into a well-established and routinised organisation.

#### Subtheme 4: Unhappiness among patients

Most of the participants reported that patients who receive little to no guidance about the availability of integrated services become frustrated, as they have to wait for a long time in queues.

One participant said that:

‘Patient gets angry to nurses as they stay longer in the queues, which make them unhappy … in some situation, you will suddenly find yourself into conflict with a patient, even [*while*] you are trying to make something clear, as they are already angry.’ (P11, 46 years, female, registered nurse)

Another one added:

‘A lot of complaints arise from patient as others stay longer in the room, and this result of chaos in the clinic by confronting nurses.’ (P7, 33 years, female, senior registered nurse)

Pescheny et al.’s^22^ findings support this study, that is, the lack of a shared understanding of integrated health services and pathways among stakeholders, including prescribers, navigators, service users and service providers, was identified as a barrier to the implementation and delivery of integrated health services.

### Theme 3: Improvement measures for the implementation of integrated services

This section will present the participants’ suggestions regarding what they wished could be done differently to improve integrated services in Namibia. The four subthemes which emerged from this theme were: train nurses who work in PHC; recruit more nurses; procure more clinical equipment and request that the region and district provide additional support and supervision.

#### Subtheme 1: Train nurses who work in primary health care

Some participants felt that the implementation of integrated services is not well initiated, or there is insufficient training, so they suggested that in the future, the government should ensure that nurses get the necessary training regarding the integrated services well before sending them out into the field. Currently, there are unnecessary delays in patient care and poor delivery of services.

One participant said that:

‘They should change the strategy of training nurses on this service; instead of training once in a year, it must be at least twice. I feel like supervisors at the regional and district level must do monthly support visits to see if intergraded service method works best for that facility.’ (P3, 34 years, female, senior registered nurse)

Another participant added:

‘Service providers should all be trained in all programmes to provide holistic care to patients.’ (P14, 40 years, male, registered nurse)

The recommendations from a study by Watt et al.^24^ concur with current study findings, suggesting that continuous training and specialisations are needed to improve providers’ skills. Moreover, Pescheny et al.^22^ found that a lack of, improper and/or inconsistent training is a barrier to rendering integrated health services, while proper training facilitates the delivery of integrated services. A study in Mozambique showed that inadequate training leads to the inefficient delivery of integrated health services.^29^

#### Subtheme 2: Recruit more nurses

The study participants suggested that the Ministry of Health and Social Services should provide more nurses so that service can be delivered to patients quickly and they can avoid keeping patients for so long.

One participant reported:

‘The Ministry is requested to improve on the shortage of nurses so that we can cover all our patients as we can help each other by implementing … integrated services.’ (P7, 33 years, female, senior registered nurse)

Another nurse added:

‘Additional staff are required to allow us to work more collaboratively and to deliver appropriate interventions and to work in more effective ways, rather than a crisis-driven focus.’ (P12, 35 years, female, senior registered nurse)

This recommendation is similar to that of Pescheny et al.’s^22^ studies in Swaziland and South Africa that revealed that a shortage of staff is a barrier to the provision of integrated health services by health care providers.^19^ Furthermore, Watt et al.^24^ also recommended that teams of care providers can only provide good care if the number of staffs is deemed sufficient in relation to the services that need to be rendered. An adequate staff-to-patient or staff-to-task ratio is important to maintain safety, hygiene and effectiveness.

#### Subtheme 3: Procure more clinical equipment

Participants suggested an increase in the provision of equipment to the facility in order to reduce the problem of overcrowding at the points of service, thereby reducing waiting times while others are using equipment.

One participant recommended:

‘The Ministry must provide equipment as is the beginning of it, because there is no way you can integrate when some of the rooms do not have beds and other equipment, as in some cases the bed used for examining sick patients is also used for palpating pregnant women.’ (P5, 30 years, female, registered nurse)

Another participant added:

‘This service is done at one site or area; therefore, there is a need for the Ministry to provide enough equipment to each clinic for integrated health service in order to improve its implementation.’(P2, 31 years, female, registered nurse)

The above findings are in line with those of Sandelowsky et al.,^30^ who recommended that health managers and policymakers allocate significantly more resources to improving service delivery. Similarly,^19^ posed that a lack of supplies and equipment is a barrier to the provision of services by health care providers.

#### Subtheme 4: Region and district to provide support and supervision

The participants suggested that additional involvement and visits by health officials to health facilities are likely to yield tangible outcomes.

One participant said that:

‘Having the officials visiting at the clinic regarding the implementation of integrated service once or twice in a year will be better, because they will have a platform on which all the challenge they are facing are revealed.’ (P10, 38 years, male, registered nurse)

Another one said:

‘The participants noted that the support, commitment and regular visits by personnel from the regional or district level on behalf of the ministry may contribute positively towards the delivery of quality integrated health services.’ (P3, 34 years, female, registered nurse)

The recommendations of this study regarding the involvement of health programme managers are similar to those of Busetto et al.,^31^ who stated that leadership and organisational culture are essential underlying characteristics that often critically influence the extent to which other inputs (collaboration, proactiveness, trained personnel and appropriately used resources) work well within integrated services. This reflects a growing recognition of the importance of effective governance in the design and implementation of health systems interventions.

### Limitations, strengths and areas for future research

This study had one limitation, namely that it was limited to urban PHC facilities situated within one district in the Kavango East region, Namibia. This limits the generalisation of the findings to other districts and regions. One major strength of this study is that the data were collected from participants who were all professional nurses with experience in PHC nursing and who had been working in PHC clinics for a minimum of two years, which enabled them to provide rich data that enhanced the trustworthiness of the study. This was also the first study to be conducted in the Namibian context on the subject; thus, it provides baseline data for integrated care programmes and future research. A mixed study comprising all PHC clinics in the region may be useful in providing more information regarding barriers to the implementation of integrated care in both urban and rural PHC clinics.

## Conclusion

This study aimed to explore and describe the facilitators and barriers to the implementation of integrated health services in Rundu health district, Namibia. The findings of this study identified barriers related to human resources, essential resources and unhappiness among patients owing to time constraints. Facilitators identified in this study include accessibility of the guidelines on integrated service and their facilitation of continuity of care. The Ministry of Health and the relevant health divisions and subdivisions, in particular the PHC division, need to address the identified shortcomings to ensure that the future delivery of integrated health services will be successful. It is of paramount importance that all stakeholders in the integrated health services domain work collaboratively toward the common goal of having integrated services be fully implemented. The findings from this study will hopefully create awareness and understanding about the facilitators and barriers to the implementation of integrated services when it comes to urban PHC in the Namibian urban context. In addition, it provides recommendations to improve the implementation of integrated services in Namibian PHC clinics.
